# The Baetylus Theorem—the central disconnect driving consumer behavior and investment returns in Wearable Technologies

**DOI:** 10.4236/ti.2016.73008

**Published:** 2016-07-28

**Authors:** James A. Levine

**Affiliations:** Obesity Solutions, Mayo Clinic, 13400 E Shea Blvd, Scottsdale, AZ 85259 and Arizona State University, Obesity Solutions, 1000 S. Cady Mall 144, Tempe, Arizona 85287

**Keywords:** Wearable technology, Consumer behavior, Investment, Entrepreneurship, Physical activity

## Abstract

The Wearable Technology market may increase fivefold by the end of the decade. There is almost no academic investigation as to what drives the investment hypothesis in wearable technologies. This paper seeks to examine this issue from an evidence-based perspective. There is a fundamental disconnect in how consumers view wearable sensors and how companies market them; this is called The Baetylus Theorem where people believe (falsely) that by buying a wearable sensor they will receive health benefit; data suggest that this is not the case. This idea is grounded social constructs, psychological theories and marketing approaches. A marketing proposal that fails to recognize The Baetylus Theorem and how it can be integrated into a business offering has not optimized its competitive advantage. More importantly, consumers should not falsely believe that purchasing a wearable technology, improves health.

## 1. Introduction

Wearable technologies are currently a $1 billion business and estimated to grow more than five-fold in the next few years. Overall, the wearable market is expected to increase substantially. A Transparency Market Research report estimated that the world wearable market was, $750 million in 2012 and expected it to increase to, $5.8 billion by 2018. Some consider that and under-estimate; Juniper Research predicted that worldwide wearable technology market would grow from $1.4 billion in 2013, to $19 billion by 2018. The market is primed for expansion.

At the same time that the market thirsts for expansion, there is a rapidly growing array of wearable sensor technology to fuel consumer, and thus business, opportunities. Examples include: MEMs chips, smart fabrics, nanotechnology, easily programmable onboard interfaces and inexpensive Integrated Circuit design [1-6]. The availability of low cost technology along with the ease of electronics design (e.g. inexpensive fabrication and decreased software prices) enables new devices to be easily and inexpensively made. Universities around the world support Innovation and Biobusiness start-up so that many graduates are entering the entrepreneurship and business-development pipeline [7-10] further fueling the expansion of wearable technologies. Noting these factors, the rate of growth of wearable technology start-ups is outstripping the rate of growth of business investment. Nonetheless, the investment hypothesis is to show a positive return. The wearable market with a many-fold expansion and multi-billion-dollar valuation is attractive for investment funds – but there are pitfalls.

Data suggest that investment strategies in wearable technologies are actually high-risk ventures because investment opportunities are outpacing the rate of growth of consumer demand; both are growing but the consumer demand at a slower growth rate [11-13]. In order to favor the investment hypothesis and reduce the investment risk in ‘wearables’, it is important to understand that there is a critical disconnect in how consumers purchase wearable technologies and how companies market them

Understanding this critical disconnect - The Baetylus Theorem – alerts consumers to adjust their expectations, enables investment risk to be minimize and lastly, The Baetylus Theorem affords business opportunities itself. In this paper the Baetylus Theorem is explained in order to drive more profitable investment decisions in wearable sensors.

## 2. The Baetylus Theorem - The critical disconnect in the wearable technology business

The Baetylus was a mythical stone in Greek Mythology - when a person held it, they were magically heeled. The Baetylus Theorem is: Consumers falsely believe that when they buy a wearable technology it will improve their well-being or health. The critical disconnect is that buying a sensor does not influence health or wellbeing. For example Thompson and colleagues provided wearable activity sensors to 49 adults AND offered them an advice book. After 24 weeks the subjects' activity levels had not changed [14]. A person's health or wellbeing is influenced by the actions/behaviors they undertake (e.g. taking a walk) or avoid (e.g. stopping smoking) not by objects they buy. Wearables do not change a person's health/wellbeing; all they do is provide feedback on some variable oftentimes with questionable accuracy. The Baetylus Theorem is that a fundamental disconnect exists whereby consumers falsely believe that buying a wearable technology to benefit them. Companies exploit the Theorem to entice consumer spending. Understanding The Baetylus Theorem on one hand should serve as a warning to consumers but on the other hand, it directly drives the successful investment hypothesis – a positive ROI.

The Baetylus Theorem is specific to Wearables and untrue for other types of biotechnology investment. Consider a standard biotech startup in health. A scientist discovers growth inhibitor, called, Chemical X, to treat breast cancer. Chemical X is tested on a petri dish and then in animals to see whether it kills breast cancer cells and tumors in animals. If so, Chemical X is then tested for safety in people and a patent is applied for. The regulatory environment (e.g. U.S. Food and Drug Administration (FDA) and the European Medicines Agency (EMA)) demands that before Chemical X can be sold as a pharmaceutical, its effectiveness is proven. Carefully regulated clinical trials occur over several years to prove the efficacy of Chemical X, after which Chemical X goes to market. In the marketplace the consumer than pays for a product that has a proven track record of improving that individual's health/well-being. This carefully regulated system for drug development does not exists for consumer Wearables.

In consumer Wearables, a very different standard exists. A scientist discovers that a health variable (e.g. exercise) is important for improving human health (e.g. exercise decreases cardiovascular disease) and conducts many scientific studies to prove this (examples: [15-17]). This scientist then argues that an important part of an exercise program is self-monitoring (examples [18-20]). Thus far, it is an evidence-based argument. The scientist then builds and patents a device for self-monitoring physical activity. If the labeling on the device was true it might say, “buying or wearing this device has not been proven to be associated with any health benefit.” However, the device is marketed in keeping with The Baetylus Theorem where consumers believe – without proof - that by buying the ‘scientific’ device, their health will improve. In fact, most people who buy physical activity trackers stop wearing them at an exponential rate [21]. Investors are not worried about how wearables are utilized with respect to improving health. Investors and companies are more are interested in selling units; an unused Wearable is just as profitable as one in use.

## 3. Why does The Baetylus Theorem occur whereby a consumer falsely believes that buying a Wearable will confer benefit?

### 3.1. Magical health-promoting objects are a part of society's collective consciousness

References to magically heeling objects - stones, clothes, arms, books, musical instruments, chariots, substances, vessels, foods & jewelry - are scattered across cultures and history. The Baetylus was a rock in Greek Mythology that conferred healing; similar objects appear in Buddhist, Jewish, Nordic, Medieval, Polynesian, Pagan, Slavic and Uralic writings. The notion that there are ‘secret’ objects that improved health is deeply embedded in the world mythology and thus the collective consciousness [22, 23].

### 3.2. C.R. Snyder's Hope Theory

The second reason The Baetylus Theorem has proven to be the critical driver of wearable sales is because of it is embedded in human psychology. The psychologist C.R. Snyder's Hope Theory linked ‘hope’ [24] to the existence of a definable goal, combined with a specific plan for reaching that goal – this leant on Adler's position that humans have goal-seeking as a central drive [25]. Health and well-being are goals of most people linked to their drive to live. And in implicit in Snyder's Hope Theory is that healthiness is an attainable goal that can be reached through an organized process of goal setting. Especially amidst consumerism, ‘buying something’ fulfills one of the steps in Snyder's model as to how people behave. It is an achievable step towards health.

### 3.3. Wilcox's Theory of Vicarious Goal Fulfillment

In the context of Wearables, Wilcox's Theory of Vicarious Goal Fulfillment [26] suggests that by buying a Wearable a person's brain acknowledges the purchase as a healthful goal. As a consequence the brain rewards the person with a similar ‘dose’ of well being as if they just completed a 5 Km run (even though all they did was make a purchase!) The consumer feels good by simply buying the sensor – as if they had just exercise.

Combining Wilcox's Theory with Snyder's Hope Theory creates a power consumer model. A person buys a Wearable as a step towards hope and the brain rewards the person as if they had actually completed a healthy behavior. The consumer has made a purchase but the psychological response is equivalent to winning a Gold Medal for nonexistent achievement! Overall, there are deep-rooted psychological reasons as to why The Baetylus Theorem is so powerful at driving consumer-sales of Wearables. Even though no health benefit is gained by buying a Wearable, a consumer feels that they have taken a meaningful step towards health through their purchase.

### 3.4. Terror Management Theory [TMT]

Terror management theory (TMT) reflects a fundamental concept in marketing theory. TMT exploits the basic psychological conflict whereby a person has a desire to live but realizes that death is inevitable [27]. The concept of TMT is derived from Becker's work, The Denial Of Death, where Becker argues the most human action is taken to avoid the inevitability of death. This is leveraged in many marketing campaigns. Car advertisements, for example, often combine the issues of family (longevity) with the threat of mortality; in one advertisement, a father drives home to reach his family and in so doing has to drive along dangerous winding roads. The advertisement captures the essence of paternity and masculinity (signals of longevity) plus the avoidance of death. TMT is used widely across marketing and perfectly aligns with selling of Wearables - as Wearables link the notion of avoiding mortality with enhancing life. Wearable Technology businesses are therefore perfect vehicles for TMT marketing approaches - avoiding mortality through purchase.

The reason the Baetylus Theorem is critical for understanding consumer behavior with respect to Wearables. The Baetylus Theorem - where a person buys a wearable sensor with the delusion that the purchase alone will confer healthfulness or happiness- is embedded in culture, psychology and marketing practices. A Wearable Technology business that fails to harness The Baetylus Theorem will underachieve in the marketplace as it ignores a primary driver of consumerism.

## 4. The Baetylus Theorem: Worked examples

### 4.1. Example 1

We convened a panel of six professionals, three scientists in three serial entrepreneurs. We presented to them 34 business concepts in health technologies and money to fund two of these business concepts. Half of the business ideas had a scientific foundation and a half of them did not. We found that the panelists consistently preferred the business propositions that were best directed towards successfully marketing to consumers – regardless of the lack of validity. Both the scientists and serial entrepreneurs devalued scientific evidence and human trial data in the presence of well-positioned marketing plans. It was clear that consumer-based positioning was more palatable in a business financing competition than accurate information and well-executed science.

### 4.2. Example 2

Consider the companies Gruve® Technologies Inc. and Fit Bit® Inc. Both companies were formed in about 2007 and both used the same technology to measure physical activity. Both patents protecting elements of the inventions and both were being launched in the United States. Two similar companies with similar missions launched in 2007: Gruve® Technologies Inc. did not succeed financially; Fit Bit® became a $4 billion company. What was the reason two such similar companies had such diverse outcomes? Fit Bit® was positioned around The Baetylus Theorem; even the name of the device implies that ‘fitness’ comes with the device. The packaging and marketing both imply healthfulness. In contrast, Gruve® Technologies focused on medical validation and carefully controlled marketplace language. Companies that are most successful selling Wearables to consumers are those that understand how The Baetylus Theorem drives ROI.

## 5. How can understanding The Baetylus Theorem drive the investment hypothesis?

The investment hypothesis states that there should be a positive Return On Investment [ROI] and that good knowledge can mitigate against risk and improve the likelihood of a positive ROI.

There are standard concepts that are used to increase the chances of a positive ROI and these are not unique to Wearable Technologies. Examples include: (a) Current market excitement. (b) Timing. (c) First movement in the consumer space. (d) Proprietary technology (issued patents, novel marketing strategies, confidential algorithms, novel data processing techniques, or exclusive license agreements). (e) Newness. (f) Reasonable accuracy. (g) Physical object. Purchasing a tangible object differentiates the Wearable Technology domain from another poorly differentiated business investment space - that of, apps. (h) The presence of a decent business backbone. These are examples of standard checkboxes that an investor might use to support an investment in Wearable Technology businesses.

### 5.1. The Baetylus Theorem influences investment strategy in Wearable Technology in specific fashion

An investor might ask first; has the company wasted research funds? The investor might ask, “Have prior investment funds been invested in a costly research based evaluation?” Scientific validation does not add value to a Wearable Technology investment. Thus, large amounts of money have been incorrectly invested in scientific research these funds could have been, (A) spent elsewhere to advance the business proposition, (B) it suggests that the company leadership has not understood the concepts that underpin The Baetylus Theorem and this is contrary to achieving a positive ROI and, (C) the business may be ethically obligated to report research findings that do not match a successful business enterprise.

Second, does the company understand Snyder's Hope Theory? The Wearable Technology business proposition should understand that the, “business pitch” should offer Hope to the consumer in the specific context described by Snyder. Hope offered by the Wearable should link the Wearable to the existence of an ‘attainable goal’ and position itself as being part of that plan to reach this goal. For example, activity sensors need to be associated with a perceived goal of health and fitness and position themselves as a tool critical to achieve that goal. In another context, a wearable sensor that detects dehydration could be linked to initiate a specific hydration plan/fluid. If the business proposition doesn't link the Wearable towards a goal that engenders hope, it is unlikely to thrive in the marketplace.

Third, does the company understand Vicarious Goal Fulfillment? Recall that Vicarious Goal Fulfillment provides the brain with a reward signal despite no actual healthful behavior having been changed. In the context of building a successful business plan for Wearable Technologies, it is critical that the marketing strategy embrace the idea that by buying the sensor an individual will reward himself or herself mentally as if having taken a step towards improved physical well being. Although it is a subtle concept it is not difficult to translate to the marketplace. For example, a marketing company could promote a device that improves posture in the following way, “Congratulations, you've just brought a device that will give you years of potential back pain relief.” This marketing strategy influences in the consumer's brain with the notion of a reward [“Congratulation”] for a behavior not actually changed. By buying the device, the consumer has not changed their posture but nonetheless gets a psychological reward for simply spending their money.

Lastly, does the company understand Terror Management Theory (TMT)? A market strategy that does not take advantage of TMT is not optimally positioning itself. TMT addresses the perpetual conflict of a human; namely, that living is the perpetual avoidance of death. This can easily be leveraged in selling a Wearable. For example, people undertaking dangerous skiing maneuvers or skydiving are used to advertise wearable video capture devices. This links the Wearable to vitality and mortality in one image. In the healthcare market, selling devices in diabetes often using the complications of diabetes and the implicit threat of death as marketing strategies.

In order to most favorably position a new Wearable Technology business for investment not only do the standard investment checkboxes need to be adhered to but there also needs to be an understanding of The Baetylus Theorem. A wearable sensor that fails to recognize the importance of The Baetylus Theorem is a company that has not optimized itself for the best return on investment.

## 6. Direct business investment opportunities that exploit The Baetylus Theorem

By understanding The Baetylus Theorem several business opportunities present themselves.

### 6.2. Example One

Anticipating the unveiling of the delusion. As time goes on the consumer base will understand that they have been seduced to buy objects that offer more hope than value. This will ultimately precipitate the development of a new regulatory environment. For instance, this has started to occur in the area of health supplements. The regulatory environment for Wearables will include elements such as: Common data motifs, international standards of validation, common scientific standards, common bio safety standards and common personal information protection standards. Each of these elements represent potentially business opportunities in of themselves, considering the value of the ‘Bluetooth®’ of wearable sensors.

### 6.2. Example Two

The collective consciousness will see that purchasing Wearables has consistently offered false hope; therefore, opportunities will exist to deliver true hope whereby the Wearable actually confers benefit. An example would be the marketing of a behavioral strategy that integrates a physical activity monitor to improving health and fitness. Behavioral-based strategies and the scalability of these are a potentially powerful business opportunities because of the volume of Wearables being sold.

## 7. Conclusion

The Wearable Technology market is anticipated to increase fivefold by the end of the decade. There will be many investment opportunities. In part because of the simplicity of building lightweight wearable technologies it is difficult to differentiate and sift through the investment opportunities. There is a fundamental disconnect in how consumers view wearable sensors; this is called The Baetylus Theorem where people believe (falsely) that by buying a wearable sensor they will receive benefit. This idea is grounded not only in the collective consciousness of society but also in established psychological constructs. A business offering that fails to recognize The Baetylus Theorem and how it can be embraced in a business offering has not optimized their competitive advantage. Health professionals must educate consumers that Wearables do not change a person's health, people's behavior does.
